# A composite six bp in-frame deletion in the melanocortin 1 receptor (*MC1R*) gene is associated with the Japanese brindling coat colour in rabbits (*Oryctolagus cuniculus*)

**DOI:** 10.1186/1471-2156-11-59

**Published:** 2010-07-01

**Authors:** Luca Fontanesi, Emilio Scotti, Michela Colombo, Francesca Beretti, Lionel Forestier, Stefania Dall'Olio, Séverine Deretz, Vincenzo Russo, Daniel Allain, Ahmad Oulmouden

**Affiliations:** 1 DIPROVAL, Sezione di Allevamenti Zootecnici, University of Bologna, Via F.lli Rosselli 107, 42123 Reggio Emilia, Italy; 2 INRA, UMR1061 Unité de Génétique Moléculaire Animale, Université de Limoges, 87060 Limoges Cedex, France; 3 UR967, Génétique Expérimentale en Productions Animales, INRA, 17700 Surgères, France; 4 UR631, Station d'Amélioration Génétique des Animaux, INRA, 31326 Castanet Tolosan, France

## Abstract

**Background:**

In the domestic rabbit (*Oryctolagus cuniculus*), classical genetic studies have identified five alleles at the *Extension *locus: *E*^*D *^(dominant black), *E*^*S *^(steel, weaker version of *E*^*D*^), *E *(wild type, normal extension of black), *e*^*J*^(Japanese brindling, mosaic distribution of black and yellow) and *e *(non-extension of black, yellow/red with white belly). Sequencing almost the complete coding sequence (CDS) of the rabbit *MC1R *gene, we recently identified two in-frame deletions associated with dominant black (c.280_285del6; alleles *E*^*D *^or *E*^*S*^) and recessive red (c.304_333del30; allele *e*) coat colours. It remained to characterize the *e*^*J*^allele whose phenotypic effect is similar to the *Orange *and *Sex-linked yellow *loci of cat and Syrian hamster.

**Results:**

We sequenced the whole CDS in 25 rabbits of different coat colours including 10 Japanese and 10 Rhinelander (tricolour) rabbits and identified another 6 bp-in frame deletion flanked by a G > A transition in 5' (c.[124G>A;125_130del6]) that was present in all animals with Japanese brindling coat colour and pattern. These mutations eliminate two amino acids in the first transmembrane domain and, in addition, cause an amino acid substitution at position 44 of the wild type sequence. Genotyping 371 rabbits of 31 breeds with different coat colour this allele (*e*^*J*^) was present in homozygous state in Japanese, Rhinelander and Dutch tricolour rabbits only (except one albino rabbit). Rabbits with *e*^*J*^/*e*^*J *^genotype were non fixed at the non-agouti mutation we previously identified in the *ASIP *gene. Segregation in F_1 _and F_2 _families confirmed the order of dominance already determined by classical genetic experiments with a possible dose effect evident comparing *e*^*J*^/*e*^*J *^and *e*^*J*^/*e *animals. *MC1R *mRNA was expressed in black hair skin regions only.

**Conclusions:**

The c.[124A;125_130del6] allele may be responsible for a MC1R variant determining eumelanin production in the black areas. However, the mechanism determining the presence of both red and black hairs in the same animal seems more complex. Expression analyses of the c.[124A;125_130del6] allele suggest that *MC1R *transcription may be regulated epigenetically in rabbits with the Japanese brindling phenotype. Further studies are needed to clarify this issue.

## Background

Coat colour in mammals is determined by the presence, distribution and biochemical activity of the melanocytes, which are specialized cells where eumelanins (black/brown pigments) and pheomelanins (yellow/red pigments) are synthesized. More than 300 loci have been shown to affect coat colour in mice regulating or altering melanocyte development and migration during embryogenesis, melanocyte morphology and functions, its components and its enzymatic machinery [1,2]. However, *Extension *and *Agouti *are the main loci that affect the production and relative amount of these two melanin types in the melanocytes [3]. These loci show epistatic interactions and usually wild type *Extension *is required for *Agouti *expression. Dominant alleles at the *Extension *locus produce black pigmentation, whereas recessive alleles extend the production of pheomelanins, causing yellow/red/pale pigmentation. On the contrary, dominant *Agouti *alleles determine pheomelanic phenotypes whereas recessive alleles cause black coat colour with a few exceptions.

The *Extension *locus encodes the melanocortin 1 receptor (MC1R) [4]. This protein belongs to the seven transmembrane G protein coupled receptors that binds the α melanocyte-stimulating hormone (αMSH) inducing eumelanin synthesis. The *Agouti *locus encodes the agouti signaling protein (ASIP) that is a small paracrine signalling protein (131-135 amino acids in different mammals) [5]. ASIP affects pigmentation blocking the αMSH-MC1R interaction which, in turn, causes a switch in pigment type from eumelanins to phaeomelanins [6,7].

Mutations of the *MC1R *gene associated with different coat colours have been described in several species (mice [4], humans [8], guinea pigs [9], cattle [10-12], pigs [13,14], horses [15], sheep [16], goats [17], dogs [18,19], foxes [20,21], bears [22], felids [23], pocket and beach mice [24,25], squirrels [26], chickens [27], Japanese quails [28], bananaquits [29], guinea fowl [30] and reptiles [31]) in which gain of function mutations produce black/dark coat colour, whereas loss of function mutations usually cause yellow/red coat colour.

**Figure 1:**
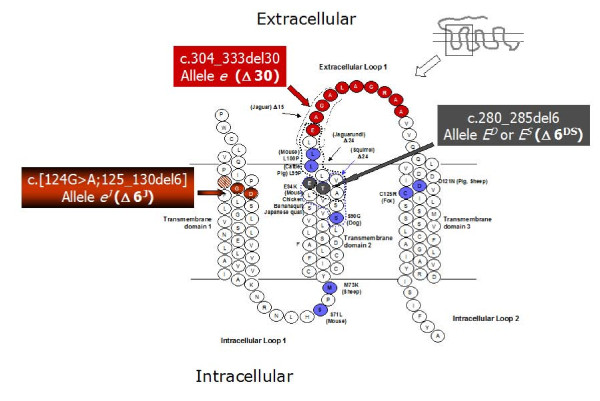
**2D structure of the rabbit MC1R protein with indicated the positions of the two in-frame deletions already described **[35]** and of the new mutation associated with the Japanese brindling coat colour**. The two and ten amino acids shaded in grey and red are those that are deleted in the rabbit c.280_285del6 (*E*^*D *^or *E*^*S*^) and c.304_333del30 (*e*) alleles, respectively [35]. The two amino acids shaded in red and black are those deleted in the newly identified c.[124A;125_130del6] allele (*e*^*J*^). The red hatched is substituted in the *e*^*J *^allele. The *E*^*D *^or *E*^*S*^, *e *and *e*^*J *^alleles have been also indicated as Δ6^DS^, Δ30, Δ6^J^, respectively. Circles bordered by a thick black line and/or coloured in blue represent the activating amino acids substitutions that have been previously reported to be associated to dominant eumelanic phenotypes in several animal species [4,10,13,16,20,27-29]. The deletions observed in jaguar, jaguarundi and squirrel [23,24] have been surrounded by a black dotted, a black bold dotted and a blue bold dotted line, respectively. The inset at the right top shows the secondary structure diagram of the whole protein with evidenced the region enlarged below it.

**Figure 2:**
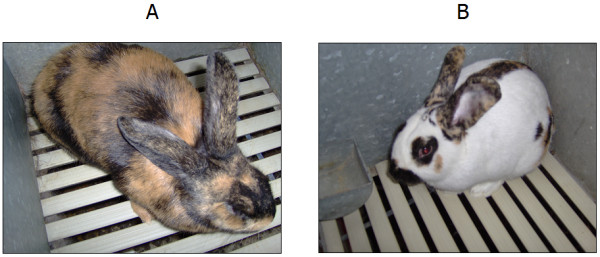
Japanese (A) and Rhinelander (B) rabbits

In the domestic rabbit (*Oryctolagus cuniculus*), classical genetic studies involving crossbreeding experiments among breeds with different coat colours have identified five alleles at the *Extension *locus: *E*^*D *^(dominant black), *E*^*S *^(steel, weaker version of *E*^*D*^), *E *(wild type, normal grey or normal extension of black), *e*^*J*^(Japanese brindling, mosaic distribution of black and yellow) and *e *(non-extension of black, yellow/red with white belly) [3,32-34]. The order of dominance is the following: *E*^*D *^>*E*^*S *^>*E *>*e*^*J *^>*e*, with possible partial dominance of *E*^*D *^over *E*^*S *^and of *E*^*S *^over *E *[3,32-34]. Sequencing almost the complete coding sequence (CDS) of the rabbit *MC1R *gene, we recently identified two in-frame deletions associated with dominant black (c.280_285del6; alleles *E*^*D *^and/or *E*^*S*^) and recessive red (c.304_333del30; allele *e*) coat colours, respectively [35]. The dominant black deletion eliminates two amino acids in the second transmembrane domain (Figure 1). We could not clarify if this deletion corresponds to the *E*^*D *^or *E*^*S *^allele as sequencing and genotyping in a large number of rabbits across 30 breeds/strains did not identify any other mutation associated with dominant black (and/or steel). Therefore it could be hypothesized the existence of only one dominant black allele and the presence of modifier genes might be the reason of different intensities of black (steel) of the two putative alleles identified by classical genetic studies. The recessive red deletion eliminates 10 amino acids of the first extracellular loop (Figure 1) and was in homozygous state in all yellow/red rabbit breeds and strains (Burgundy Fawn, Gold Saxony, New Zealand Red, and Thuringian breeds and yellow/red animals of other breeds not fixed for any coat colour) [35]. It remained to characterize the *e*^*J *^(Japanese brindling) allele. The Japanese brindling coat colour variety probably appeared in France within the second half of the 19th century and subsequently was introduced in England and other countries [36,37]. The Japanese brindling pattern can be defined as a yellow coat mottled with black, therefore this coat colour seems determined by the presence of two different types of melanocytes in different skin areas, one producing eumelanin and another one producing pheomelanin [3,33,36,37]. The *e*^*J *^allele should be fixed in the Japanese and Rhinelander breeds [33,34]. The former breed (named after its coat colour) has the classic mottled phenotype (Figure 2A) that is similar to the tortoiseshell pattern observed in female cats and female Syrian hamsters. The coat colour in these two species is caused by the *Orange *and *Sex-linked yellow *loci, respectively, that are two non-homologous X chromosome loci [38,39]. The coat colour phenotype determined by the rabbit *e*^*J *^allele is also similar to that determined in guinea-pig by the *e*^*p *^(tortoiseshell) allele at the *Extension *locus described by classical genetic studies [40,41] but not characterized at the molecular level, yet [9]. The Rhinelander rabbit breed has a tricolour pattern with black, yellow and white areas (Figure 2B). The white regions are caused by the absence of mature melanocytes due to a dominant *English spotting *locus allele [3,32,33,35,42]. The coloured patches are much larger than those of the tortoiseshell-like pattern probably because a reduced number of melanoblasts in the skin allows for a spatial expansion of the melanoblast-derived clones. This tricolour pattern is similar to the calico coat colour in cat determined by the epistatic interaction between the *Spotting *and *Orange *loci [38] and in guinea-pigs to the effect of the white-spotting factor *s *in *e*^*p*^*/e*^*p *^animals [3]. An *Extension *allele with similar (but not overlapping) phenotypic effect to the rabbit *e*^*J *^allele has been described in pig [14]. In this species the *E*^*p *^allele, that is caused by a frameshift mutation (a 2 bp insertion) in the *MC1R *coding sequence, determines red coat colour in Hereford, Linderöd and Tamworth pigs but frequent somatic reversions re-establish the correct reading frame and produce black coat colour in skin areas determining a spotted phenotype in a red background. As a matter of fact, epistatic effects of the *Dominant white/KIT *locus on this *Extension *allele might determine the white and black spotted phenotype or the complete white colour of some pig breeds [14,43].

Here we investigated the *MC1R *gene in Japanese and Rhinelander rabbits and identified a novel in-frame deletion associated with the *e*^*J *^coat colour. In addition we analysed in these rabbits the *MC1R *gene expression in skin specimens of different coloured regions.

## Results

### Rabbit *MC1R *gene sequences

In our previous study we did not completely sequence the CDS of the rabbit *MC1R *gene and for some breeds the *Extension *allele status was obtained by genotyping the two in-frame deletions identified and not by sequencing [35]. Here we amplified and sequenced 1348 bp (considering a wild type sequence) of the *MC1R *gene (the whole CDS of 954 bp, 14 bp of the 5'-untranslated region and 380 bp of the 3'-untranslated region) in 25 rabbits of different breeds including 10 animals of the Japanese and 10 animals of the Rhinelander breeds. The sequences obtained from Burgundy Fawn and Thuringian rabbits confirmed the presence of the 30 bp in-frame deletion (c.304_333del30) as the determinant of the *e *allele (EMBL accession number FN658678). The c.280_285del6 was identified in a Checkered Giant rabbit carrying the *E*^*D *^(or *E*^*S*^) allele confirming what we previously reported [35] (EMBL accession number FN658677). Surprisingly, another 6 bp deletion flanked by a G>A transition in 5' (c.[124G>A;125_130del6]), was identified in all Japanese and Rhinelander sequenced rabbits (EMBL accession number FN658679). The 6 bp deletion included 2 nucleotides of codon 42, three nucleotide of codon 43 and one nucleotide of codon 44. Together with the G>A transition this 6 bp deletion eliminates two amino acids in the first transmembrane domain (D and G at positions 42 and 43 of the wild type sequences) and, in addition, causes an amino acid substitution at position 44 (p.L44T), considering the wild type sequences (Figure 1). EMBL accession numbers of the wild type sequences (obtained from Giant Grey and Blue Vienna rabbits; see [35]) are FN658675 and FN658676.

### Genotyping in different rabbit breeds

**Figure 3:**
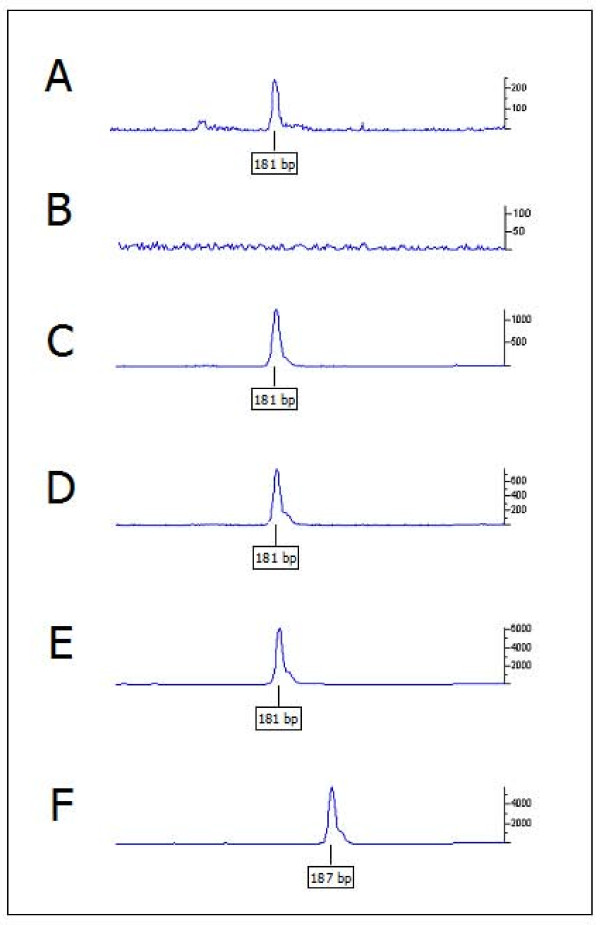
**Electropherograms showing the amplified *MC1R *fragments obtained from different sources**. *MC1R *product amplified from A) cDNA obtained from a black hair skin region of a Rhinelander rabbit, B) cDNA obtained from a red hair skin region of a Rhinelander rabbit (the same result was obtained in white hair skin regions), C) genomic DNA isolated from a black hair skin region of a Rhinelander rabbit, D) genomic DNA isolated from a red hair skin region of a Rhinelander rabbit, E) genomic DNA isolated from blood of a Rhinelander rabbit, F) genomic DNA isolated from blood of a Checkered Giant rabbit. No amplification was obtained in B. The Rhinelander rabbit was homozygous for allele c.[124A;125_130del6] (*e*^*J*^) and the Checkered Giant rabbit was homozygous for allele c.280_285del6 (*E*^*D *^or *E*^*S*^). The size of the amplified fragments is reported in the boxes below the electrophoretic peaks.

In order to confirm the putative role of this new allele in determining the Japanese and tricolour phenotypes we genotyped by fragment analysis the c.125_130del6 mutation in a total of 371 rabbits belonging to 31 breeds. Genotyping data are reported in Table 1 (see Figures 3E and 3F as examples of the genotyping results). These animals were also genotyped for the other two in-frame deletions associated with the *e *and *E*^*D *^(or *E*^*S*^) alleles [35]. All rabbits of the Japanese, Rhinelander and Dutch tricolour breeds were homozygous for the new composite mutation. This mutation was also identified: 1) in heterozygous condition with the c.280_285del6 allele, in two black and white Checkered Giant rabbits belonging to a colony in which there was segregation of the tricolour and black and white phenotypes; 2) in heterozygous condition with a wild type *Extension *allele in one Giant Grey rabbit (with wild type coat colour); 3) homozygous in one Angora albino rabbit. The presence of this mutation in other animals that do not have the Japanese or tricolour phenotype demonstrates the dominance of the c.280_285del6 and of the wild type *Extension *alleles over the c.[124A;125_130del6] allele, confirming the results of classical genetic studies [1,33,36,37]. In addition, albino rabbits do not express any other coat colour locus allele due to its status caused by a mutation in the tyrosinase (*TYR*) gene [44]. All rabbits were also genotyped for the insertion in the *ASIP *gene (c.5_6insA) we recently identified to be the causative mutation of the nonagouti black coat colour [45]. Interestingly, all three possible genotypes were identified in the Japanese and Rhinelander rabbits whereas 7 Dutch tricolour rabbits were homozygous for the c.5_6insA mutation and one was heterozygous. To exclude the presence of additional *ASIP *mutations we sequenced the three coding exons and parts of the intronic regions in two Japanese or Rhinelander animals for each c.5_6insA genotype. No other polymorphisms were identified apart those already reported [45].

**Table 1:** Genotypes of the novel *MC1R *6 bp composite deletion (c.[124G>A;125_130del6] or Δ6^J^) in rabbits of different breeds

Breed (no. of animals)	**Coat colour (no. of animals)**^**1**^	Genotypes^2,3^		
		**181/181**	**181/187**	**187/187**

Alaska (7)	self black	-	-	7
Angora (1)	albino-white	1	-	-
Belgian Hare (3)	reddish laced with black	-	-	3
Blanc de Hotot (3)	white with black markings	-	-	3
Blue Vienna (39)	dark blue	-	-	39
Burgundy Fawn (15)	fawn	-	-	15
Bristle White (3)	bristle white	-	-	3
Californian (43)	white with black markings	-	-	43
Champagne d'Argent (39)	silver as surface colour and dark blue as under-colour	-	-	39
Checkered Giant (29)	white with black markings	-	2	27
Checkered Small (7)	white with black (6) or blue (1) markings	-	-	7
Coloured Dwarf (5)	bristle white (2), hare-grey (1), Havana (1), chinchilla (1)	-	-	5
Dutch (17)	with black markings (9*), tricolour (8**)^4^	8**	-	9*
Ermine (1)	white with blue eyes	-	-	1
Fairy Marburg (1)	grey-light blue	-	-	1
Fairy Pearly (3)	pearling grey	-	-	3
Giant Chinchilla (21)	chinchilla	-	-	21
Giant Grey (12)	wild-grey	-	1	11
Giant White (7)	white albino	-	-	7
Havana (2)	dark brown	-	-	2
Japanase (32)	Japanese brindling	32	-	-
Lop (8)	wild-grey (7); with Madagascar markings (1)	-	-	8
Lop Dwarf (3)	wild-grey (2); self black (1)	-	-	3
Mini Silver (4)	black with silvering	-	-	4
New Zealand White (33)	white-albino	-	-	33
Rex (1)	black dalmatian	-	-	1
Rhinelander (11)	white with black and yellow markings (tricolour)	11	-	-
Silver (10)	black with silvering	-	-	10
Tan (2)	black fire	-	-	2
Thuringian (3)	shaded yellow/brown	-	-	3
White Vienna (6)	white-blue eyes	-	-	6

Total (371)		52	3	316

^1 ^When rabbits with different coat colours were sampled in a breed, the corresponding number of analysed animals is reported.

^2 ^The genotypes are indicated according to the obtained amplified fragments in bp: 181 = fragment with the 6 bp composite deletion (c.[124A;125_130del6] allele indicated also as Δ6^J^); 187 = normal fragment, that could be derived from the *E, e *and *E^D^*or E^S^ alleles. The number of animals with the three genotypes is reported.

^3 ^These animals have been genotyped for the c.280_285del6 and c.304_333del30 deletions of the *MC1R *gene [35] and for the c.5_6insA deletion in the *ASIP *gene causing the black nonagouti coat colour [45].

^4 ^For the Dutch breed, asterisks (* or **) have been included to link their coat colour description to the genotyping results.

### Family based analyses and colour segregation

Three F_1 _families were created crossing animals of different breeds (Additional file 1). All parental animals were homozygous for the c.5_6insA *ASIP *mutation. The first family was obtained by crossing a Rhinelander buck with a Thuringian doe homozygous for the c.304_333del30 mutation (*e *allele). Of the 7 obtained F_1 _rabbits, 2 had the Japanese coat colour pattern (but with predominance of the yellow/red colour) and 5 were similar to the buck (tricolour) even if, again, they showed a predominance of the yellow/red spots over the black spots. This confirms the dominance of the c.[124A;125_130del6] allele over the *e *allele and that there was a dose effect of the new mutation that was in heterozygous condition in the F_1 _animals. A second F_1 _family was created by crossing the same Rhinelander buck with a Japanese doe obtaining 9 F_1 _rabbits. Four of them had the same coat colour pattern of the buck and 5 were like the doe. In this family, the tricolour or Japanese brindling phenotypes were determined by the segregation of a dominant *English spotting *allele (heterozygous in the Rhinelander father), like in the previous family [3,32,33,35,42]. The third F_1 _family was constituted by 10 F_1 _rabbits obtained by crossing a Checkered Giant buck (homozygous for the c.280_285del6 allele) and the Japanese doe of the previous family. All F_1 _animals were completely black (no. = 3) or black and white (no. = 7). Again, the black and white F_1 _rabbits were determined by a dominant *English spotting *locus allele, heterozygous in the Checkered Giant buck [3,32,33,35,42]. This family confirms the dominance of the c.280_285del6 allele over the c.[124A;125_130del6] allele. F_2 _animals were obtained by crossing two F_1 _rabbits of this family. The F_1 _animals were carriers of the c.[124A;125_130del6] *MC1R *allele and of the *English spotting *locus allele. Of the nine F_2 _rabbits, 4 were completely black (two were homozygous for the c.280_285del6 allele and two had the heterozygous genotype c.280_285del6/c.[124A;125_130del6]), 3 were black and white (one was homozygous for the c.280_285del6 allele and two had the heterozygous genotype c.280_285del6/c.[124A;125_130del6]), 2 had a tricolour phenotype, confirming the segregation of the Japanese brindling coat colour together with homozygosity for the c.[124A;125_130del6] allele in these latter rabbits (data not shown).

### *MC1R *gene expression in different skin regions

Several skin specimens were sampled from two rabbits (one Rhinelander rabbit and one Japanese solid rabbit) just after moulting. Skin samples were collected from regions with different hair colours (white, black and red). Total RNA was isolated, retrotranscribed and amplified to evaluate (as a simple fragment size analysis) the presence of *MC1R *transcripts including the c.125_130del6 mutation (see Methods). In all skin samples with black hair a transcript with expected size was identified whereas no amplification was obtained from skins of both red or white hair regions (Figures 3A and 3B). A control glyceraldehyde 3-phosphate dehydrogenase (*GAPDH*) cDNA fragment was amplified from all skin samples (Additional file 2). Amplification of DNA extracted from the same skin samples used for RNA analysis showed the expected *MC1R *gene fragment (with the c.125_130del6 mutation) (Figure 3).

## Discussion

According to the results we obtained, the c.[124G>A;125_130del6] composite mutation is associated with the *e*^*J *^allele at the rabbit *Extension *series. This is the third in-frame *MC1R *mutation associated with a coat colour in rabbit, making this species quite unique. One *MC1R *allele determined by a nonframeshift deletion associated only with melanism has been observed in each of other four species (jaguar, jaguarundi, squirrel and guinea fowl) [23,26,30].

The c.280_285del6 rabbit allele (*E*^*D *^or *E*^*S*^), that eliminates two amino acids in the second transmembrane domain), may determine a constitutive activation of the MC1R protein, that in turn, drives eumelanin production. It seems that the *e*^*J *^in-frame mutation that eliminates two amino acids in the first transmembrane domain (including an amino acid substitution at position 44) could cause the melanic phenotype of the black skin areas by either determining constitutive activation of the receptor or increasing the affinity to αMSH. Otherwise, the presence of black spots or black hairs in Rhinelander and Japanese rabbits could not be explained. The black of the Japanese and tricolour rabbits has been also suggested to be dominant by the pioneering studies of Castle [36] and Punnett [37]. The nonagouti insertion [45] is not the causative mutation of the black areas in these animals for the following reasons: i) in our population survey, all three genotypes for the c.5_6insA *ASIP *mutation [45] were identified in Japanese and Rhinelander rabbits, but their coat colour did not apparently differ; ii) as the *Extension *locus should be epistatic over the *Agouti *locus, the presence of at least a wild type allele is needed to show the allelic status at the *Agouti *locus, and the *e*^*J *^allele seems clearly a non wild-type allele, at least in our crossbreeding experiments; iii) classical genetic studies indicated that the presence of any *Agouti *allele does not alter the black in the Japanese rabbits [36,37]. It could be also possible that (an)other not yet identified mutation(s) in the promoter region of the *ASIP *gene can determine the black phenotype in rabbits not homozygous for the c.5_6insA *ASIP *mutation but this hypothesis is quite remote as we did not have any evidence of the presence of other non-agouti mutations in a survey that included 31 rabbit breeds [45]. According to classical genetic studies [36,37] and to our results, the *e*^*J *^allele is dominant (or partially dominant) over the *e *allele but recessive over the other alleles of the *Extension *series. Another example of recessive melanism determined by an in-frame deletion in the *MC1R *gene has been recently reported in guinea fowl [30]. However, to obtain a direct experimental proof of the effects of the *e*^*J *^in-frame composite deletion on the receptor activities and interactions in rabbit pharmacological studies should be carried out.

It also remains to be explained the presence of red coat colour areas associated with this mutation. The effect of the rabbit *e*^*J *^allele resembles the mosaicism of the sex linked *Orange *locus in female cat and that of *Sex linked yellow *in female Syrian hamster [38,39]. The Lyon hypothesis for random X inactivation [46] can explain the two sex linked phenotypes in heterozygous animals carrying both a wild-type allele and a mutated allele. However the rabbit *Extension *is an autosomal locus and the mosaic phenotype is present in homozygous *e*^*J*^/*e*^*J *^animals (or in *e*^*J*^/*e *rabbits, in which the *e*^*J *^allele is dominant). Komai [47,48] suggested that the Japanese brindling coat colour in rabbit could be determined by both *E*^*D *^and *e *alleles that are present on the same chromosome as a result of unequal crossing-over. Searle [3] re-interpreted Komai hypothesis [47,48] from the point of view of dosage compensation as Lyon [46] looked at the situation of X-linked loci. Of the two alleles (*E*^*D *^and *e*) on the same chromosome only one can be active in each chromosome so as to achieve the correct gene dosage. As the *E*^*D *^is dominant, all cell types in which at least a copy of *E*^*D *^would be active (in one or the other chromosome) produce black hair, whereas the cells in which all *E*^*D *^are not active (that phenotypically appear *e*/*e*) would produce yellow/red hair. The result would be a brindled effect with black predominating over red. In case of heterozygous *e*^*J*^/*e *this hypothesis would predict that half the cells give black hair and half red hair, but as inactivation is random there could be *e*^*J*^/*e *rabbits that are completely or almost completely red, as reported by Castle [36] and Punnett [37]. According to our results, the Komai-Searle hypothesis cannot be confirmed. The *e*^*J *^allele is different from both the *E*^*D *^and *e *alleles that are caused by distinct nonframeshift deletions.

Another hypothesis on the presence of autosomal variegation was suggested by Chase [49] who indicated that this phenotype in *e*^*p*^/e^*p *^guinea-pigs could be the result of unstable genes. Somatic mutations to black could occur in yellow mutants. In pigs, a high frequency of somatic reversion of a frameshift mutation in the *MC1R *gene cause black spotting in a red background [14]. This reversion re-establishes the correct reading frame in a *MC1R *transcript that includes another missense mutation (p.D121N) associated with the dominant black coat colour [13]. However, the evidence obtained for the rabbit Japanese brindling coat colour may indicate that the mechanism determining this phenotype is different from what was observed in pigs. In pigs, the 2 bp mutation determining the *E*^*p *^allele causes a frameshift mutation in the CDS sequence of the pig *MC1R *gene producing a transcript that includes a premature stop at codon 56 whereas the rabbit *e*^*J *^allele is determined by a nonframeshift deletion that could be predicted to maintain the MC1R action. In pigs, gene expression evaluation in red skin spots showed the presence of the mutated *MC1R *transcript only (including the 2 bp insertion) whereas two *MC1R *transcripts, one with the insertion of 2 bp and one reversed (without this insertion), were detected in black skin spots [14]. This was not unexpected since partial expression of the non mutated transcript should be sufficient to restore the dominant black coat colour in pigs [14]. In Japanese and Rhinelander rabbits, only one *MC1R *transcript (having the 6 bp deletion of the *e*^*J *^allele) was observed in black hair skin regions whereas red regions did not express any *MC1R *transcript (at least at the level of sensitivity of our qualitative RT-PCR). However, the *MC1R *gene (including the 6 bp deletion of the *e*^*J *^allele) was amplified from DNA isolated from black and red skin regions indicating that somatic disrupting mutations did not occur in pheomelanic regions. Even if these results were obtained in a few animals, based on these evidences it could be possible to suppose that a regulatory mechanism, driven by another mutation in linkage disequilibrium with the c.[124G>A;125_130del6] composite mutation or by the same composite mutation, may determine the Japanese brindling phenotype in rabbits. To identify a promoter polymorphism we sequenced about 1 kb of the *MC1R *gene 5'-flanking region in two Japanese and in two Checkered Giant rabbits but we did not find any sequence difference (data not shown). Regulation of *MC1R *expression might be epigenetically determined as already reported for other genes [50,51]. Constitutive methylation of the *MC1R *promoter could inhibits *MC1R *expression causing red coat colour. On the other hand, DNA hypomethylation in some cellular skin clones could lead *MC1R *expression that might determine black coat colour. In this case, as also similarly hypothesized in pigs, since the expression of one of the two copies is sufficient to restore a dominant black coat colour [14], this mechanism could act by chance in one or the other gene promoter of the diploid somatic skin cells. A dose-effect mechanism that could confirm this hypothesis was evident in the *e*^*J*^/*e *tricolour rabbits (in which the *e *allele produces a non functional MC1R receptor [35]) obtained crossing a Rhinelander buck with a Thuringian doe (Additional file 3). In these rabbits there is a preponderance of the red spots over the black spots compared to tricolour *e*^*J*^/*e*^*J *^rabbits. A similar observation has been reported by Castle [36] and Punnett [37] who even showed that, as an extreme phenotype, some *e*^*J*^/*e *rabbits did not have black hair. It is worthwhile to mention that the data in our hands cannot formally exclude the possibility that the Japanese coat colour is caused by a frequent somatic mutation in a regulatory region that re-establishes the *MC1R *gene expression. However, this mechanism cannot completely explain the Japanese brindling in solid coloured rabbits. These animals could have striped-like patterns of black and red as well as brindle-like phenotypes in specific body regions for which a directional epigenetic activation or silencing could be a plausible explanation instead of a random somatic mutation reverting the phenotype in clone-derived cell lines, as in the case of the *E*^*p *^allele in spotted pigs.

## Conclusions

We have identified a novel composite mutation in the rabbit *MC1R *gene associated with the Japanese brindling coat colour phenotype that is a mixture of yellow/red and black hairs. In the case of the epistatic interaction with another locus (*English spotting*), it assumes the tricolour pattern with well defined red and black spots. This mutation might be responsible for a dominant (over allele *e*) or recessive (over other *Extension *alleles) eumelanin production in the black hair skin regions. However, the mechanism(s) that cause(s) the presence of both red and black hairs in the same animal seems more complex. Results we obtained about the expression of the *e*^*J *^allele indicate that a regulatory mechanism, driven by another mutation in linkage disequilibrium or by the same identified composite mutation, could determine the brindling phenotype. Epigenetics could be involved in determining this coat colour phenotype. Other studies are needed to evaluate this hypothesis.

## Methods

### Animals and samples

Blood samples were collected from 10 unrelated Japanese, 10 unrelated Rhinelander and one Burgundy Fawn, one Thuringian, one Checkered Giant, one Giant Grey and one Blue Vienna rabbits registered to the breed herdbook (all these animals were used for sequencing as reported below). Hair root samples were also obtained from: 22 Japanese, 1 Rhinelander and 8 Dutch tricolour rabbits. Three F_1 _families were constructed crossing 1) a Rhinelander buck with a Thuringian doe (obtaining 7 F_1 _rabbits), 2) the same Rhinelander buck with a Japanese doe (obtaining 9 F_1 _rabbits) and 3) a Checkered Giant buck (with blue spots) with the same Japanese doe (obtaining 10 F_1 _rabbits). Two F_1 _rabbits of the latter family were crossed producing one F_2 _family (9 rabbits). Blood samples were collected from all these animals after slaughtering at slaughterhouse and stored at -20°C. Pictures were taken from all F_1_, F_2 _and parental rabbits. Skin samples were collected at the slaughterhouse from one Japanese and two Rhinelander rabbits slaughtered just after moulting. These samples were obtained from completely red, black and, in Rhinelander rabbits, also from completely white hair skin regions. In the Japanese rabbit used for skin sampling it was possible to identify completely red and completely black hair regions. Skin specimens were immediately snap frozen in liquid nitrogen and stored at -80°C.

Hair root samples were also collected from additional 315 rabbits belonging to other 29 different breeds (including the Dutch breed, for which animals with other coat colours were sampled) (Table 1). All these rabbits were registered to their breed herdbooks.

### Sequencing of the *MC1R *and *ASIP *genes

DNA was extracted from blood and skin samples using the Wizard^® ^Genomic DNA Purification kit (Promega Corporation, Madison, WI) or using rapid extraction methods for hair roots [35,52]. Primers for rabbit *MC1R *amplification and sequencing (Additional file 4) were already reported [35] or newly designed using PRIMER3 (Whitehead Institute for Biomedical Research, Cambridge, MA) on consensus gene regions obtained aligning the *MC1R *gene in different species or obtained using rabbit trace record sequences belonging to the *MC1R *gene identified by BLASTN analysis. Primers for *ASIP *sequencing (the three coding exons and part of intronic regions) were previously reported [45]. *MC1R *gene sequences were obtained from the 10 Japanese and 10 Rhinelander sampled rabbits and from one Burgundy Fawn, one Thuringian, one Giant Grey, one Vienna Blue and one Checkered Giant rabbits already genotyped for the previously detected in-frame deletions [35,52]. *ASIP *gene sequences were obtained for two Japanese or Rhinelander rabbits for each c.5_6insA *ASIP *genotype, as previously determined [45]. PCR was performed using a TGradient thermal cycler (Biometra, Goettingen, Germany) or a PT-100 thermal cycler (MJ Research, Watertown, MA, USA) in a volume of 20 μL containing 10-100 ng DNA template, 1 U DNA EuroTaq DNA polymerase (EuroClone Ltd., Paington, Devon, UK), 1× PCR Buffer, 2.5 mM dNTPs, 10 pmol of each primer and optimised MgCl_2 _concentrations (from 2.0 to 2.5 mM). PCR profile was as follows: 5 min at 95°C; 35 amplification cycles of 30 s at 95°C, 30 s at 60/65°C, 30 s at 72°C; 5 min at 72°C. For sequencing, 3-5 μL of PCR product was treated with 2 μL of ExoSAP-IT^® ^(USB Corporation, Cleveland, Ohio, USA) following the manufacturer's protocol. Cycle sequencing of the PCR products was obtained with the Big Dye v3.1 kit (Applied Biosystems, Foster City, CA, USA) and sequencing reactions, after a few purification steps using EDTA 0.125 M, Ethanol 100% and Ethanol 70%, were loaded on an ABI3100 Avant sequencer (Applied Biosystems). All sequences were visually inspected, edited, assembled, and aligned with the help of the BioEdit software v. 7.0.5.2 http://www.mbio.ncsu.edu/BioEdit/bioedit.html and the CodonCode Aligner software http://www.codoncode.com/aligner.

### Genotyping

Analysis of the novel 6 bp deletion was obtained by amplification of a portion of the *MC1R *gene (Additional file 4) and by capillary electrophoresis for fragment analysis. Genotyping of this mutation was carried out using DNA samples extracted from blood or hair root samples collected from the 371 rabbits of different breeds and from the rabbits of the three F_1 _and F_2 _families. Genotypes were also obtained from DNA extracted from skin samples. All these animals were also genotyped for the other two in-frame *MC1R *deletions associated with the *e *and *E*^*D *^(or *E*^*S*^) alleles already identified [35,52] and by the *ASIP *insertion associated with the black nonagouti coat colour we recently reported [45]. To analyse the novel 6 bp deletion, PCR profile was as described above and as reported in Additional file 4. Amplification was obtained in a final volume of 10 μL with the forward primer labelled with 6-FAM at 5' (Additional file 4). Amplified products were loaded on a ABI3100 Avant capillary sequencer (Applied Biosystems). For this analysis 1-2 μL of reaction product was diluted in 10 μL of Hi-Di formamide (Applied Biosystems) and added to 0.1 μL of Rox labelled DNA ladders (500HD Rox, Applied Biosystems). Labelled DNA fragments were sized using GeneScan v. 3.7 and Genotyper v. 3.7 software (Applied Biosystems).

### RNA extraction and RT-PCR

Isolation of total RNA from rabbit skin specimens (about 100 mg) was carried out using the RNeasy^® ^Lipid Tissue kit (Qiagen) following the manufacturer's instructions. After RNA extraction, about 1 μg of total RNA was treated by RNase-Free DNase set (Qiagen) and 40 ng were retrotranscribed with Improm II Reverse Transcription system (Promega) using oligo(dT) primers and following the manufacturer's protocol. cDNA was amplified with the same primers used for genotyping the 6 bp novel *MC1R *deletion as reported above and in Additional file 4. Two-three μL of amplified fragments were loaded in a ABI3100 Avant capillary sequencer (Applied Biosystems) as described above. *GAPDH *cDNA amplification was used as reference (Additional files 2 and 4). *GAPDH *fragments were electrophoresed in 10% polyacryamide:bisacrylamide 29:1 TBE 1× gels and visualized with 1× GelRed Nucleid Acid Gel Stain (Biotium Inc., Hayward, CA, USA). PCR fragments of the *MC1R *gene obtained with non labelled primers were also confirmed by sequencing as described above.

## Authors' contributions

LuF conceived the study, analysed sequences and obtained data, contributed to the sampling and to the construction of rabbit families, coordinated and organized the laboratory work and drafted the manuscript. ES reared the rabbit families, genotyped and collaborated in the RT-PCR analyses. MC performed the RT-PCR experiment. FB and LiF sequenced and genotyped. SD, DA and SDe collected samples. DA, VR supervised the work and were involved in the design of the study. AO co-conceived the study, contributed to the sampling and to drafting the manuscript. All authors reviewed the manuscript and accepted the final version.

## Supplementary Material

Additional file 1 **Three F_1 _rabbit families obtained crossing animals of different breeds with different genotypes at the *Extension *locus**. Family 1 is obtained crossing a Rhinelander buck with a Thuringian doe, Family 2 is obtained crossing a Rhinelander buck with a Japanese doe, Family 3 is obtained crossing a Checkered Giant buck with a Japanese doe. The c.280_285del6 (*E*^*D *^or *E*^*S*^), c.304_333del30 (*e*), and c.[124A;125_130del6] (*e*^*J*^) alleles have been indicated as Δ6^DS^, Δ30, Δ6^J^, respectively. The *MC1R *genotypes of the parental animals is reported together with the genotype and coat colours of the F_1 _rabbits. All parental animals were homozygous for the nonagouti mutation identified in the *ASIP *gene [45]. The Checkered Giant buck of family 3 is homozygous for a recessive *Dilute *locus allele (*d*) determining the blue coat colour [3,32,33]. Click here for file

Additional file 2 ***GAPDH *cDNA fragment amplified from retrotranscribed RNA extracted from skin specimens**. Lines A and B: from black hair skin regions of a Rhinelander rabbit. Lines C and D: from red hair skin regions of a Rhinelander rabbit. Line E: control genomic DNA. Line F: DNA ladder. Click here for file

Additional file 3 **Tricolour rabbits with Δ6^J^/Δ30 (A) and Δ6^J^/Δ6^J ^(B) genotypes**. Rabbits with these two genotypes differ in terms of extension of black regions. Click here for file

Additional file 4 **Primers and PCR conditions**. Primer sequences, PCR conditions and use of the reported primer pairs. Click here for file
