# Supplementary material for: A composite six bp in-frame deletion in the melanocortin 1 receptor (MC1R) gene is associated with the Japanese brindling coat colour in rabbits (Oryctolagus cuniculus)
Source: BMC Genet. 2010 Jul 1;11:59. doi: 10.1186/1471-2156-11-59 (PMC3236303; doi:10.1186/1471-2156-11-59)

Additional file 1 –Three F_1 rabbit families obtained crossing animals of different breeds with different genotypes at the *Extension* locus.

The c.280_285del6 (E^D or E^S), c.304_333del30 (*e*), and c.[124A;125_130del6] (e^J) alleles have been indicated as $\Delta6^{DS}$, $\Delta30$, $\Delta6^J$, respectively. The genotypes of the parental animals is reported together with the genotype and coat colours of the F_1 rabbits. The Checkered Giant buck of family 3 is homozygous for a recessive *Dilute* locus allele (*d*) determining the blue coat colour [3,31,32].

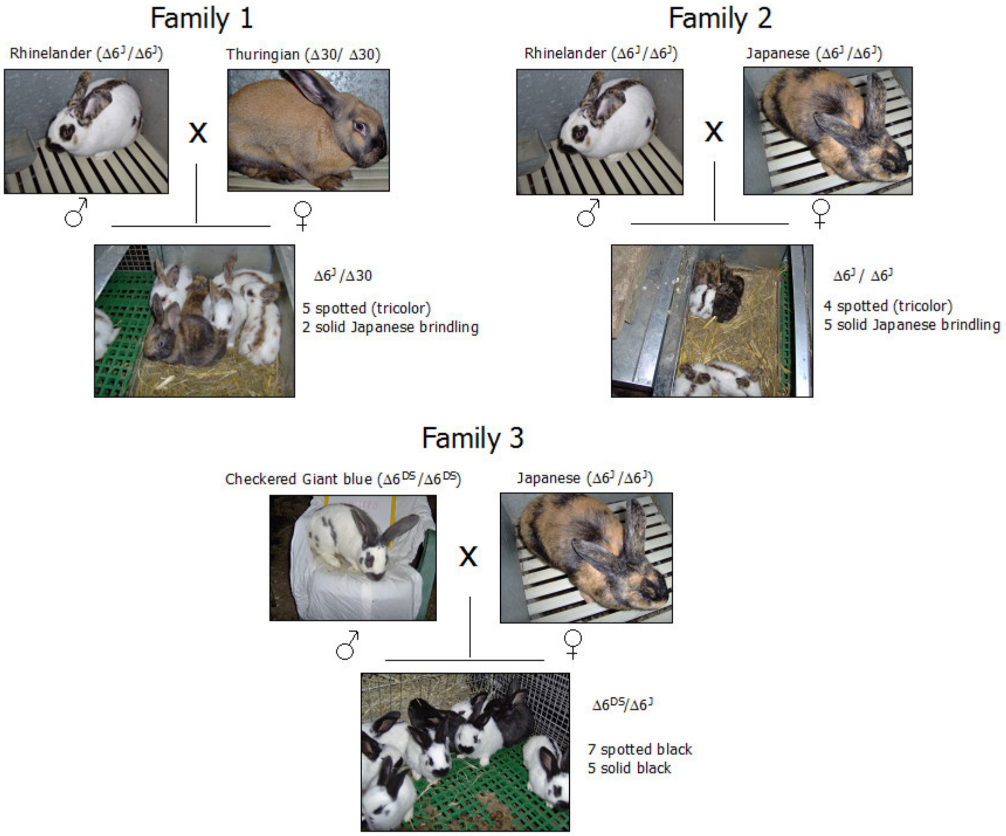

Supplement: Additional file 1 — Three F1 rabbit families obtained crossing animals of different breeds with different genotypes at the Extension locus. Family 1 is obtained crossing a Rhinelander buck with a Thuringian doe, Family 2 is obtained crossing a Rhinelander buck with a Japanese doe, Family 3 is obtained crossing a Checkered Giant buck with a Japanese doe. The c.280_285del6 (ED or ES), c.304_333del30 (e), and c.[124A;125_130del6] (eJ) alleles have been indicated as Δ6DS, Δ30, Δ6J, respectively. The MC1R genotypes of the parental animals is reported together with the genotype and coat colours of the F1 rabbits. All parental animals were homozygous for the nonagouti mutation identified in the ASIP gene [45]. The Checkered Giant buck of family 3 is homozygous for a recessive Dilute locus allele (d) determining the blue coat colour [3,32,33]. [file 1471-2156-11-59-S1.PDF]
