# Supplementary material for: A composite six bp in-frame deletion in the melanocortin 1 receptor (MC1R) gene is associated with the Japanese brindling coat colour in rabbits (Oryctolagus cuniculus)
Source: BMC Genet. 2010 Jul 1;11:59. doi: 10.1186/1471-2156-11-59 (PMC3236303; doi:10.1186/1471-2156-11-59)

Additional file 2 – *GAPDH* cDNA fragment amplified from retrotranscribed RNA extracted from skin specimens.

A and B: from black hair skin regions of a Rhinelander rabbit. C and D: from red hair skin regions of a Rhinelander rabbit. E: control genomic DNA. F: DNA ladder.

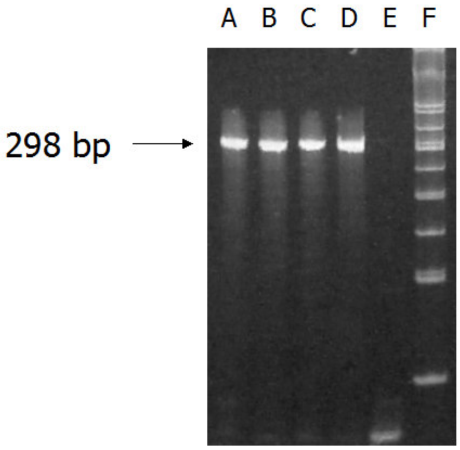

Supplement: Additional file 2 — GAPDH cDNA fragment amplified from retrotranscribed RNA extracted from skin specimens. Lines A and B: from black hair skin regions of a Rhinelander rabbit. Lines C and D: from red hair skin regions of a Rhinelander rabbit. Line E: control genomic DNA. Line F: DNA ladder. [file 1471-2156-11-59-S2.PDF]
