# Supplementary figures and images for: A composite six bp in-frame deletion in the melanocortin 1 receptor (MC1R) gene is associated with the Japanese brindling coat colour in rabbits (Oryctolagus cuniculus)
Source: BMC Genet. 2010 Jul 1;11:59. doi: 10.1186/1471-2156-11-59 (PMC3236303; doi:10.1186/1471-2156-11-59)

**Additional file 3 – Tricolour rabbits with  $\Delta 6^J/\Delta 30$  (A) and  $\Delta 6^J/\Delta 6^J$  (B) genotypes.**

A

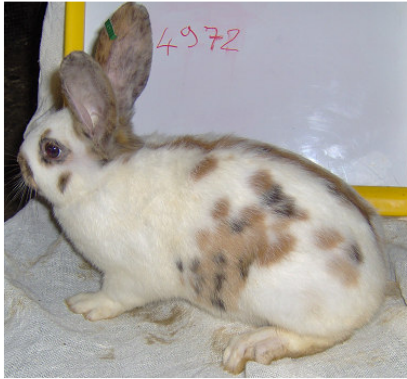

B

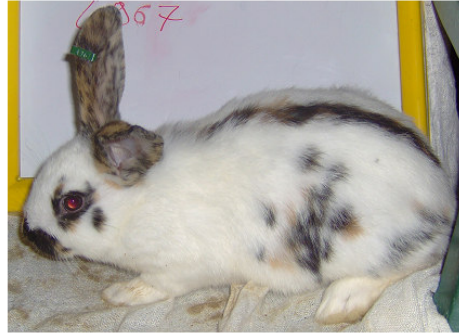

Supplement: Additional file 3 — Tricolour rabbits with Δ6J/Δ30 (A) and Δ6J/Δ6J (B) genotypes. Rabbits with these two genotypes differ in terms of extension of black regions. [file 1471-2156-11-59-S3.PDF]
