# Supplementary material for: A composite six bp in-frame deletion in the melanocortin 1 receptor (MC1R) gene is associated with the Japanese brindling coat colour in rabbits (Oryctolagus cuniculus)
Source: BMC Genet. 2010 Jul 1;11:59. doi: 10.1186/1471-2156-11-59 (PMC3236303; doi:10.1186/1471-2156-11-59)
Supplement: Additional file 4 — Primers and PCR conditions. Primer sequences, PCR conditions and use of the reported primer pairs. [file 1471-2156-11-59-S4.PDF]

Additional file 4 – Primer sequences, PCR conditions and use of the reported primer pairs.

Primer sequences, PCR conditions and use of the reported primer pairs

Primer pair name/gene	Forward and *reverse* primers (5' – 3')	PCR[2]	Product length (bp)	Use
MC1R_1[1]	CACCAGCCCCTTCCTGAT<br>*GTAGCGCAGTGCGTAGAAGA*	64/1.0	488	Sequencing
MC1R_2[1]	ACCTGCTGGTGAGCGTGA<br>*AAGAAGATGCCCAGGAGGAT*	61/1.0	502	Sequencing
MC1R_3[1]	GTCCTGCTCTGCCTCATCAT<br>*AGTCTCTGGCTGCGGAAG*	58/1.0	335	Sequencing
MC1R_4	CATCATCTGCAACTCCATCG<br>*CTGCAGCCACACCCTTCT*	62/1.0	495	Sequencing
MC1R_5	6FAM-GGGACTATGCCCATGCAG<br>*CCACTACCAGCAGGTTCTCC*	60/2.0	181/187	Genotyping and RT-PCR analysis (fragment analysis)
GAPDH	GAGCTGAACGGGAAACTCAC<br>*TGCTGTAGCCAAATTCGTTG*	59/1.0	298	RT-PCR

[1] Primers used for sequencing by Fontanesi et al. [34].
[2] PCR conditions: annealing temperature (°C)/$MgCl_2$ concentration (mM).
